# ERK5 signalling in prostate cancer promotes an invasive phenotype

**DOI:** 10.1038/sj.bjc.6606062

**Published:** 2011-01-25

**Authors:** A K Ramsay, S R C McCracken, M Soofi, J Fleming, A X Yu, I Ahmad, R Morland, L Machesky, C Nixon, D R Edwards, R K Nuttall, M Seywright, R Marquez, E Keller, H Y Leung

**Affiliations:** 1 Institute for Cancer Sciences, College of Medical, Veterinary and Life Sciences, University of Glasgow, Glasgow G61 1BD, UK; 2 Urology Research, The Beatson Institute for Cancer Research, Glasgow Centre for Cancer Research, Garscube Estate, Switchback Road, Bearsden, Glasgow G61 1BD, UK; 3 Urology Research Group, Northern Institute for Cancer Research, Medical School, University of Newcastle Upon Tyne, Framlington Place, Newcastle Upon Tyne NE2 4HH, UK; 4 School of Biological Sciences, University of East Anglia, Norwich NR4 7TJ, UK; 5 Department of Pathology, Western Infirmary, NHS Greater Glasgow and Clyde, Glasgow G11 6NT, UK; 6 WestCHEM, Department of Chemistry, University of Glasgow, Glasgow G12 8QQ, UK; 7 Departments of Urology and Pathology, Michigan University, 1500 East Medical Center Drive, 5304 CCGC, Ann Arbor, MI 48109, USA

**Keywords:** prostate cancer, signalling, invasive phenotype

## Abstract

**Background:**

Aberrant mitogen/extracellular signal-regulated kinase 5 (MEK5)–extracellular signal-regulated protein kinase 5 (ERK5)-mediated signalling has been implicated in a number of tumour types including prostate cancer (PCa). The molecular basis of ERK5-driven carcinogenesis and its clinical relevance remain to be fully characterised.

**Methods:**

Modulation of ERK5 expression or function in human PCa PC3 and PC3–ERK5 (stably transfected with ERK5) cells was performed using siRNA-mediated knockdown or the MEK inhibitor PD18435 respectively. *In vitro* significance of ERK5 signalling was assessed by assays for proliferation, motility, invasion and invadopodia. Expression of matrix metalloproteinases/tissue inhibitors of metalloproteases was determined by Q-RT–PCR. Extracellular signal-regulated protein kinase 5 expression in primary and metastatic PCa was examined using immunohistochemistry.

**Results:**

Reduction of ERK5 expression or signalling significantly inhibited the motility and invasive capability of PC3 cells. Extracellular signal-regulated protein kinase 5-mediated signalling significantly promoted formation of *in vivo* metastasis in an orthotopic PCa model (*P*<0.05). Invadopodia formation was also enhanced by forced ERK5 expression in PC3 cells. Furthermore, in metastatic PCa, nuclear ERK5 immunoreactivity was significantly upregulated when compared with benign prostatic hyperplasia and primary PCa (*P*=0.013 and *P*<0.0001, respectively).

**Conclusion:**

Our *in vitro*, *in vivo* and clinical data support an important role for the MEK5–ERK5 signalling pathway in invasive PCa, which represents a potential target for therapy in primary and metastatic PCa.

Prostate cancer (PCa) is a significant contributor to morbidity and mortality in the Western World. In 2009, there were an estimated 192 280 new cases of PCa diagnosed with an estimated 27 360 PCa deaths in the United States (Altekruse *et al*, 2010). Over the last three decades, its incidence has trebled, mostly attributed to improved detection through widespread prostate-specific antigen (PSA) testing. In addition, as life expectancy improves, the rise in incidence is anticipated to continue, worsening its health burden. Currently, treatment selection depends on the prognostic factors of Gleason grade, TNM stage and serum PSA levels, as well as the patient's age, health and own preference. Patients deemed at low risk are recommended active surveillance, but most organ-confined disease is treated with curative intent, with surgery or radiation. For locally advanced and metastatic PCa, androgen ablation therapy remains one of the commonest treatment, showing an 80–90% response rate (Ramsay and Leung, 2009). However, within 12–33 months, the disease almost invariably progresses to an androgen-independent or castrate-resistant state. After this, treatment is limited and death occurs within a few years (Ramsay and Leung, 2009). Alternative treatment is therefore required for advanced PCa.

Advances in molecular biology have increased our understanding of the numerous molecular pathways that may contribute to prostate carcinogenesis. Careful analysis of these signalling pathways may facilitate the development of targeted therapies. Recently, our laboratory showed the importance of the MEK5/ERK5 (mitogen/extracellular signal-regulated kinase 5/extracellular signal-regulated protein kinase 5) cascade in PCa (Mehta *et al*, 2003; McCracken *et al*, 2008). Extracellular signal-regulated protein kinase 5 belongs to the family of mitogen-activated protein kinases (MAPKs). The other main subfamilies are ERK1/2, c-Jun NH2-terminal protein kinases and p38 MAPKs (Chang and Karin, 2001). All are activated by extracellular stimuli such as growth factors and environmental stresses, and are recognised to control cell proliferation, migration and differentiation. Extracellular signal-regulated protein kinase 5 is the specific substrate of MEK5, and is regulated by mitogens (EGF and G-CSF), cytokines (LIF and CT-1) and stress (H_2_O_2_ and sorbitol) (Ranganathan *et al*, 2006). Downstream effectors of the ERK5 cascade are not fully defined, but include myocyte enhancement factor (MEF) 2, Sap1 and c-Myc (Wang and Tournier, 2006). Our laboratory recently proposed ERK5 to be clinically significant in PCa, its cytoplasmic expression in the primary tumour correlating with Gleason sum score (*P*<0.0001). Interestingly, significant nuclear localisation of ERK5 was strongly associated with both unfavourable patient survival outcome and the development of castrate-resistant disease (*P*<0.0001) (McCracken *et al*, 2008).

This study aimed to define the importance of ERK5 signalling in prostate carcinogenesis, by examining ERK5-mediated function by manipulation of its expression/function as well as evaluating nuclear ERK5 expression in primary and metastatic clinical PCa specimens.

## Materials and methods

### Cell culture, siRNA transfection experiments and western blotting

Cells are maintained and used as described previously: PC3, PC3-derived ERK5 overexpressing (PC3-ERK5) cells and inducible HEK293 cells expressing MEK5D (a constitutively active mutant of MEK5) (Mehta *et al*, 2003; McCracken *et al*, 2008). Extracellular signal-regulated protein kinase 5 knockdown was carried out as reported by Sharma and Goalstone (2005). Extracellular signal-regulated protein kinase 5-specific siRNA (5′-GGTGTTGGCTTTGACCTGGAGGAAT-3′) was used (Eurogentec, Southampton, UK), and all experiments were performed at 10 nM siRNA concentration. Non-silencing (or scrambled) siRNA was included as controls.

Western blot analysis was performed as described previously (Mehta *et al*, 2003; McCracken *et al*, 2008). Antibodies were used at the following dilutions: anti-ERK5 1 : 500, anti-phospho ERK5 1 : 500, p44/42 MAP kinase 1 : 1000, anti-phospho p44/42 MAP kinase (Cell Signalling Technology, Hertfordshire, UK) and anti-alpha tubulin 1 : 8000 (Santa Cruz Biotechnology, Santa Cruz, CA, USA). Horseradish peroxidase-conjugated secondary antibodies (Cell Signalling Technology) were applied at 1 : 3000 and detected using the enhanced chemiluminescence detection kit (Amersham, Piscataway, NJ, USA).

### Cell proliferation

In all, 1.5 × 10^5^ cells were seeded in six-well plates. siRNA transfection was performed and serial cell counts were taken at 24, 48, 72 and 96 h using a Casey cell counter. Each experiment was repeated at least three times, and at least three wells were used per condition each time. Where appropriate, MEK inhibitor was used in place of siRNA transfection.

### Cell tracking, chemotaxis migration and chemo-invasion assays

Cell tracking motility assays were performed 48 h post-transfection with ERK5 siRNA. PC3–ERK5 or PC3 cells were studied using a Nikon (Melville, NY, USA) TE2000 time lapse microscope ( × 20 magnification). Images were taken every 15 min over an 18 h period. Image J software (Bethesda, MD, USA) was used to track random cellular motility; accumulated and euclidean (direct) distance were measured. At least eight cells were tracked per field and three fields were viewed in each experiment which was repeated twice.

In addition, the migrative potential of PC3 cells, ±ERK5 and ±PD184352 was assessed using the chemotaxis-based BD-Falcon Transwell system (San Jose, CA, USA). Cells (8 × 10^4^) were resuspended in 500 *μ*l basal medium ±PD184352 and placed in the upper Transwell compartment. In all, 750 *μ*l of BM±EGF (100 ng ml^–1^) was placed in the lower compartment to act as a potential attractant. After 24 h incubation at 37 °C and 5% CO_2_, cells that had not migrated were removed from the upper face of the membranes with cotton buds. The filters were fixed in methanol for 30 min at −20 °C, stained with Haematoxylin Harris’ (VWR International Ltd, Leicestershire, UK) for 1 h at room temperature and then washed carefully in dH_2_O. After air dried, the membranes were mounted in DPX. The average number of cells per field of view (eight random fields per membrane) was counted under a light microscope at × 20 magnification. For chemo-invasion assays, the same protocol was employed with Biocoat Matrigel invasion chambers in duplicate (Becton Dickinson Labware, San Jose, CA, USA; cat. no.: 354480). Each experiment was repeated at least three times and each time the mean numbers of migrated or invading cells were taken from three chambers. Similarly, invasion assay with siRNA-transfected PC3 or PC3–ERK5 cells were studied as above.

### MMP promoter studies

At 48 h before transfection, 3 × 10^4^ PC3 cells were seeded per well in a 48-well plate. *Matrix metalloproteinase* (*MMP)9* (−670), *MMP2* (−1659) and *MMP1* (−512) constructs containing 5′-flanking fragments upstream to the transcription initiation start site linked to a luciferase reporter gene (MMP-luc) were co-transfected with ERK5 or empty plasmid (pcDNA3.1) using Superfect reagent, according to the manufacturer's recommendation. After 24 h, cells were serum starved and left for another 16–24 h before luciferase activities were determined. Cells were co-transfected with a *β*-gal-CMV plasmid to allow normalisation of transfection efficiency. A total of 300 ng of DNA were transfected, containing 100 ng MMP-luc, 150 ng MEK5D and 50 ng *β*-galactosidase reporter. The *MMP1/MMP2* and *MMP9* promotor constructs were obtained as kind gifts from Dr Y Sun (Parke Davies Pharmaceutical Research, UK) and Dr D Boyd (MD Anderson Cancer Centre, USA), respectively.

### Quantitative real-time RT–PCR to profile MMP and TIMP expression

Design of specific primers for human MMP and tissue inhibitors of metalloprotease (TIMP) genes and respective PCR reactions were performed as described (Altschul *et al*, 1990; Nuttall *et al*, 2003), with each PCR reaction containing 5 ng of reverse transcribed RNA in 25 *μ*l. The identity of PCR products was confirmed by direct sequencing of the amplicon. The 18S ribosomal RNA gene was used as an endogenous control to normalise for differences in the amount of total RNA in each sample, using previously validated procedures (Wall and Edwards, 2002; Nuttall *et al*, 2003). To determine the relative RNA levels within the samples, standard curves for the PCR reaction were prepared by using the cDNA from one sample and making two-fold serial dilutions covering the range equivalent to 20–0.625 ng of RNA (for 18S analyses, the range was 4–0.125 ng).

### Invadopodia assay

*In vivo* assessment of invadopodia was performed as described (Ayala *et al*, 2008). In brief, A375MM or PC3 cells were seeded onto gelatin-coated coverslips at a density of 1 × 10^4^ or 2 × 10^4^, respectively. Drug treatment groups were as follows: controls (no treatment or DMSO, 7.5 *μ*l ml^–1^), 5 *μ*M PD18435 and 15 *μ*M PD18435. For ERK5 knockdown experiments, A375 cells were transfected with either ERK5-specific or scrambled siRNA before plating onto gelatin-coated coverlips. PC3 cells were transfected with ERK5 as described previously (McCracken *et al*, 2008). After 24 h, cells were fixed for 20 min in 4% paraformaldehyde, washed three times in PBS and blocked for 10 min in NH_4_Cl. Cells were permeablised in 0.1%Triton/PBS for 4 min and then stained with 594-Phalloidin (1 : 100) for 30 min. Cells were given three final washes in PBS followed by two washes in dH_2_O and then mounted onto labelled glass slides.

A Zeiss (Thornwood, NY, USA) Axiopskop was used to capture images of at least 20 cells per test condition. For each cell, the red and green channels were used to capture the actin cytoskeleton and the gelatin layer under the cell showing any degradation, respectively. Image J and a plugin were used to evaluate these images and return a measure of degradation in terms of pixels. Two-tailed Student's *t*-test was performed on the data to establish levels of statistical significance in any differences observed.

### *In vivo* orthotopic prostate tumourigenesis study

For orthotopic tumour growth, dorso-lateral prostates of male CD-1 nude mice were injected with either PC3–ERK5 cells (stably transfected with transfected pEGFP-C1-ERK5) or the control cells (stably transfected with the empty plasmid pEGFP-C1 vector) at 2.4 × 10^6^ cells per 25 *μ*l serum free RPMI as previously described (Somers *et al*, 2003). Tumours were grown over a period of 12 weeks. The prostates were excised, weighted and fixed overnight in formalin before embedded in paraffin. All prostates were processed and cut in the same manner by a single histology technician to aid standardisation. All experiments were carried out in accordance with UK animal regulations.

### Immunohistochemical analysis

Prostate cancer tissue microarray (TMA) was supplied by the University of Michigan Prostate Specialised Programme of Research Excellence. Tissues were obtained through either prostatectomy or rapid autopsy program with appropriate Internal Review Board approval as previously described (Rubin *et al*, 2000). Tissue microarray contained benign (normal) prostate samples (*n*=30), benign prostatic hypertrophy (BPH) samples (*n*=27), prostatic intraepithelial neoplasia (PIN) (*n*=24), 80 cases of prostatic cancer as well as 32 metastatic lesions (lymph node (*n*=10), liver samples (*n*=17), lung (*n*=2) and soft tissue (*n*=3)). Full tissue sections of benign and tumour human prostate were also used for optimisation of immunohistochemistry and *in situ* hybridisation. All tissue samples were radical prostatectomy samples, collected and used according to the ethical guidelines and procedures approved by the institutional supervisory committee. Immunohistochemistry for ERK5 expression was performed based on previously described protocol (Gnanapragasam *et al*, 2003). Immunoreactivity signals were assessed by independent observers (Maria Soofi and Morag Seywright) blinded to the clinical details. Nuclear ERK5 immunoreactivity was measured on histoscore scale from 0 (no staining) to 300 (very strong staining). *In situ* hybridisation for mir143 was performed as previously reported (Clape *et al*, 2009).

### Statistical analysis

Data are shown as the mean±s.d. where possible and statistical analysis was obtained using SPSS version 15.0, computer software (SPSS Inc., Chicago, IL, USA). A *P-*value of <0.05 was taken to indicate statistical significance. Mann–Whitney test was used to test for differences of ERK5 immunoreactivity in different histological groups of prostate pathology. Chi-square test was used to assess data on metastasis in the orthotopic prostate model.

## Results

### Suppression of ERK5 function impaired the invasive capacity of PCa cells

**Figure 1:**
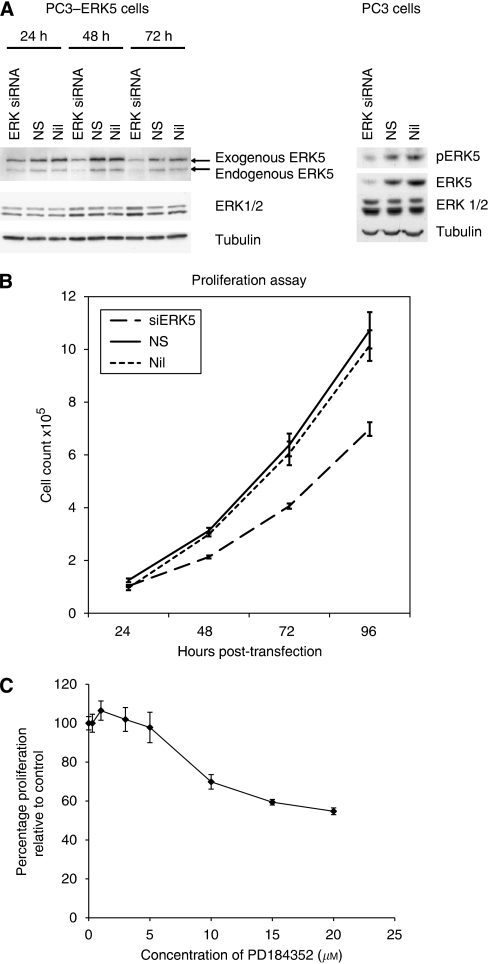
(**A**) Extracellular signal-regulated protein kinase 5 expression in PC3–ERK5 stable clone (left-hand panel) and parental PC3 cells (right-hand panel) following siRNA-mediated ERK5 directed knockdown (KD), compared with non-silencing control (NS) and sham transfection (Nil). (**B**) Proliferation assay following ERK5 KD in PC3–ERK5 cells. (**C**) Effects of a range of doses of PD184352 (MEK inhibitor) on proliferation of PC3 cells.

Successful knockdown of endogenous and exogenous ERK5 was achieved in PC3 and PC3 cells stably transfected with ERK5 (PC3-ERK5) respectively (Figure 1A). Expression of both endogenous and transfected ERK5 was significantly reduced, with evidence of suppressed pERK5 level following reduced ERK5 expression. Following transfection with siRNA targeting ERK5, suppression of ERK5 expression was sustained for at least up to 72 h, with around 70% reduction in ERK5 expression. Extracellular signal-regulated protein kinase 5 targeting siRNA was shown to be specific: throughout the duration of the experiment, ERK1/2 expression was not affected by siRNA targeting ERK5.

As ERK5 overexpression has been shown to be associated with increased proliferation *in vitro* (McCracken *et al*, 2008; Zen *et al*, 2009), we tested the effects of siRNA-mediated ERK5 knockdown on proliferation as well as motility and invasion. In PC3–ERK5 cells, a significant reduction in the rate of proliferation was observed on ERK5 knockdown, when compared with the controls (non-silencing siRNA and sham transfection controls respectively) at 96-h post-transfection (*P*<0.005; Figure 1B).

To further assess the potential mitogenic effects of ERK5 signalling in prostate carcinogenesis, the effects of suppressed endogenous ERK5 expression by siRNA or function by the MEK inhibitor PD184352 were investigated in a proliferation assay using PC3 parental cells. Contrast to the data on PC3–ERK5 cells, no convincing anti-proliferative activities was observed neither with ERK5-targeted siRNA knockdown nor with MEK inhibitor PD184352 (Figure 1C and unpublished data). Even at relatively high dose of PD184352 (up to 20 *μ*M), the reduction of proliferation has yet to reach 50% at these high doses, off-target effects are likely to contribute to the observed anti-proliferative effects. It should be noted that, in PC3 cells, PD184352 blocked phosphorylation of ERK1 at 0.3 *μ*M and ERK5 at 3 *μ*M, respectively (Supplementary Figures 1A and B), similar dose-related effects were observed in HEK293 cells (Supplementary Figure 1C).

**Figure 2:**
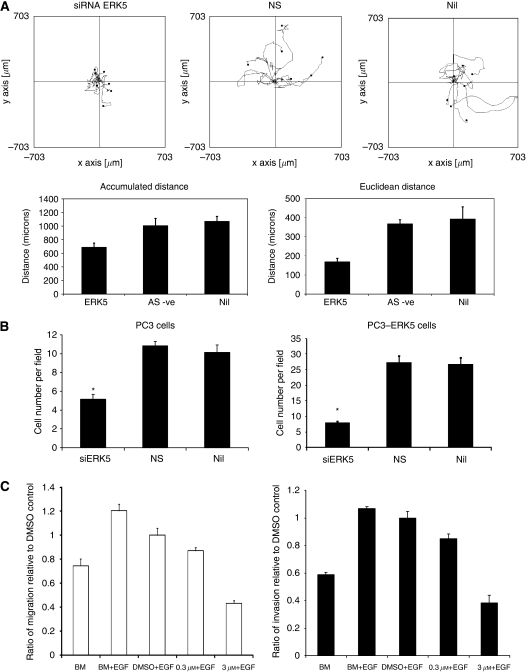
(**A**) Suppression PC3–ERK5 cellular motility by siRNA-mediated ERK5 KD (siERK5) when compared with control siRNA (NS, non-silencing) transfection or sham transfection (Nil) (^*^P<0.005). Top panel showing the ‘spider plot’ of motile pattern of tracked cells; bottom panel shows overall distance travelled (accumulated and euclidean distances). (**B**) Suppression of cellular invasion in PC3 and PC3–ERK5 cells following siRNA-mediated ERK5 KD. (**C**) PD184352 at ERK5 suppressing dose of 3 *μ*M inhibited EGF-driven cellular migration and invasion in PC3 cells.

We therefore hypothesised that ERK5 signalling may contribute to prostate carcinogenesis by driving cellular motility and/or invasion rather than proliferation. Random cellular motility was studied using real time cell tracking. Targeted knockdown of ERK5 expression by siRNA significantly decreased cell motility when compared with the non-silencing siRNA and untransfected cells. Untransfected and control transfected PC3–ERK5 cells display high levels of random motility. Both accumulated and euclidean distances following ERK5 knockdown were significantly reduced when compared with the controls (*P*<0.005; Figure 2A). Similarly, siRNA-mediated knockdown of ERK5 expression in PC3 cells reduced its motility (euclidean distance, Supplementary Figure 2). In keeping with our previous observation (McCracken *et al*, 2008), using an *in vitro* chemo-invasion matrigel assay, PC3–ERK5 cells have an enhanced invasive capability when compared with PC3 cells, with 25 (27±15) and 10 (10±4) cells per field, respectively. On transfection with ERK5-targeted siRNA, the invasive capability of PC3 cells was significantly inhibited by 2.1- and 1.97-fold when compared with control siRNA-transfected and -untransfected PC3 cells, respectively (*P*<0.005; Figure 2B). As expected, PC3–ERK5 cells are more dependent on ERK5 signalling, and showed a more dramatic suppression of cellular invasion on ERK5 knockdown, when compared with the parental PC3 cells, with a 3.5- and 3.4-fold suppression in invasion (*P*<0.005; Figure 2B).

To confirm the data from ERK5 knockdown experiments, MEK1 inhibitor PD184352 was used to suppress ERK1/2 activation alone at 0.3 *μ*M and ERK-1, -2 and -5 activities at a higher concentration of 3 *μ*M before stimulation with EGF (100 ng ml^–1^) (Mody *et al*, 2001). Assays for chemotaxis and chemo-invasion were performed. In PC3 cells, 0.3 and 3 *μ*M PD184352 significantly suppressed ERK1/2 alone and ERK-1, -2 and -5 functions, respectively (Supplementary Figures 1A and B). Inhibition of ERK1/2 alone (PD184352 at 0.3 *μ*M) did not affect EGF-mediated cellular migration and invasion in PC3 cells, but at pan ERK1/2/5 inhibiting dose of PD184352 (0.3 *μ*M), EGF-induced migration and invasion were significantly decreased (*P*<0.001, respectively; Figure 2C).

### ERK5-mediated invasive phenotype *in vitro* and *in vivo*

**Figure 3:**
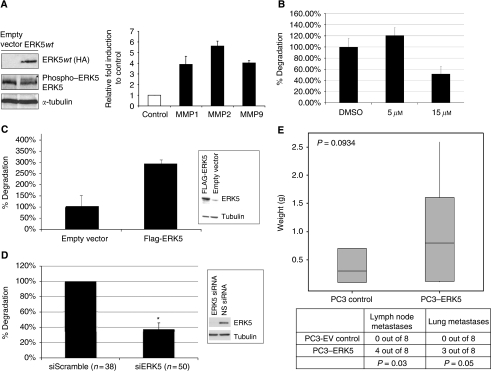
(**A**) Extracellular signal-regulated protein kinase 5-induced MMP promoter activity – (left panel) ERK5 DNA was transiently transfected into PC3 cells where a band (^*^) corresponding to phospo-ERK5 was seen on stimulation with EGF. (right panel) PC3 cells were co-transfected with MMP1, MMP2 or MMP9 promoter luciferase constructs along with ERK5 or empty plasmid. Transfection efficiency was assessed and normalised to *β*-galactosidase activity. Data represent mean fold induction compared with basal level of promoter activity from sets of quadruplicate assays±s.e. Experiments were performed three times. (**B**) Invadopodia formation by A375MM cells is significant suppressed by treatment with 15 *μ*M (but not 5 *μ*M) PD184352 (*P*<0.005). (**C**) Transfected ERK5 significantly drives invadopodia formation in PC3 cells (*P*<0.005). Insert of western blot shows upregulated ERK5 expression in PC3 cells. (**D**) Invadopodia formation in A375MM cells is significantly suppressed by siRNA-mediated knockdown of ERK5 expression (^*^P<0.05). Control cells were transfected with non-silencing scrambled siRNA. Number in brackets denotes the cells studied for each condition. Insert of western blot shows reduced ERK5 expression following siRNA transfection. (**E**) PC3–ERK5 cells form significantly more metastatic lesions (lymph nodes and lung) than control cells in an orthotopic prostate model (*P*=0.03 and *P*=0.05 respectively).

MMP promoter luciferase constructs for *MMP1*, *MMP2* and *MMP9* were studied in the presence of transiently transfected ERK5 expression in PC3 cells. Matrix metalloproteinase-1, -2 and -9 were selected as they have been previously implicated in MEK5 signalling (Mehta *et al*, 2003). On ERK5 transfection, transcriptional activities of the *MMP-1*, *-2* and *-9* promoters (corrected for *β*-galactosidase and empty vector contsrol) were induced by 3.89-, 5.61- and 4.04-fold, respectively (*P*<0.001 for all three experiments, Figure 3A). In addition, a PCR-based expression analysis for a panel of proteases was then performed using HEK293 cells stably expressing an inducible constitutively active MEK5 mutant (MEK5D-HA) with negligible background MEK5 expression (Mehta *et al*, 2003). Triplicate samples of uninduced and induced cells were analysed. Consistent with the promoter luciferase data, on activation of MEK5/ERK5 signalling, MMP2 and MMP9 mRNA expression were upregulated by 1.8- and 2.1-fold, respectively, although no increase was seen in MMP1 mRNA expression (Supplementary Table). In addition, MMP12 and TIMP2 expression were upregulated by 2.7- and 2.2-fold, respectively, with MMP16 expression reduced by 1.7-fold. We therefore confirmed *MMP2* and *MMP9* as target MMPs in MEK5/ERK5 signalling, and identified *MMP12*, *MMP16* and *TIMP2* as novel potential proteases and regulator downstream of the MEK5/ERK5 pathway. Their role in the ERK5-driven invasive phenotype warrants further investigation.

Invadopodia are proteolytically active protrusions formed by invasive tumour cells when grown on an extracellular matrix (ECM) substratum ((Ayala *et al*, 2008) and reference within). On the basis of our data on ERK5-mediated invasion and MMP expression, we hypothesised that suppression of ERK5 function by PD18435 at an ERK5 inhibitory dose will significantly suppress invadopodia formation. This is particularly relevant as ERK1/2 has been implicated to have a key regulatory input in invadopodia formation and ECM degradation (Ayala *et al*, 2008). The human malignant melanoma A375 cancer cells are well established for invadopodia assay (see Supplementary Figure 3) and were plated on gelatin and cultured for 24 h in the presence of either PD18435 (at 5 or 15 *μ*M) or the corresponding concentration of DMSO (control). A375 cells treated with 15 *μ*M PD18435 (an inhibitory dose for pan ERK1/2/5) showed significantly less gelatin degradation when compared with the vehicle control (*P*=0.0014) and ERK1/2 suppressing dose of PD18435 (5 *μ*M; *P*<0.001) (Figure 3B). Furthermore, siRNA-mediated knockdown of ERK5 in A375 cells also significantly suppressed their ability to form invadopodia (Figure 3D). PC3 cells form invadopodia much less efficiently than A375 cells, generally producing a low level of diffuse signal (Supplementary Figure 3). Hence, ERK5 was transfected into PC3 cells to test if ERK5 overexpression will promote invadopodia formation. Transfected ERK5 expression in PC3 cells significantly enhanced invadopodia formation (*P*<0.005, Figure 3C).

The *in vivo* impact of ERK5 on the invasiveness of prostate tumourigenesis was further investigated in an orthotopic prostate tumour model. PC3–ERK5 cells or the empty vector control cells were injected into the dorso-lateral prostate of nude mice (*n*=8 per group). As expected, after 12 weeks, tumour incidence in both cohorts was 100% (16 out of 16) with a statistically insignificant difference in their respective increase in tumour burden (as assessed by prostate weight) (Figure 3E). In keeping with ERK5′s role in promoting an invasive phenotype, a statistically significant increase in metastases to lymph nodes (4 out of 8 *vs* 0 out of 8) and lung (3 out of 8 *vs* 0 out of 8) was observed in the PC3–ERK5 cells when compared with control cells.

### ERK5 expression in clinical PCa

**Figure 4:**
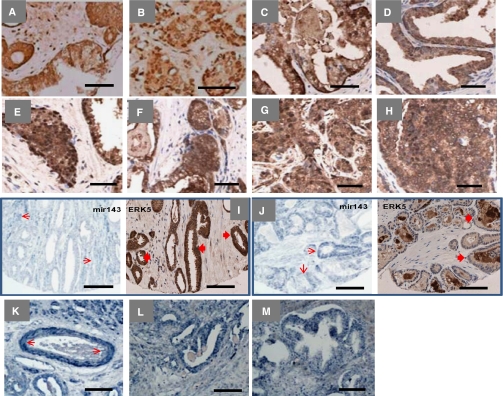
Extracellular signal-regulated protein kinase 5 protein and mir143 transcript expression in clinical prostate cancer (PCa) by immunohistochemistry (IHC) and *in situ* hybridisation (ISH) respectively. Extracellular signal-regulated protein kinase 5 immunoreactivity in (**A**) Gleason 7 PCa, (**B**) Gleason 9 PCa, (**C**) BPH, (**D**) normal prostate, (**E**) PIN, (**F**) Castrate-resistant PCa (CRPC), (**G**) liver metastasis, (**H**) lymph node metastasis; (**I** and **J**) mir143 mRNA and ERK5 protein expression analysis in sequential slides from two cases of PCa (solid and open arrows signify ERK5 protein and mir143 transcript expression, respectively), (**K**) mir143 expression in endothelium; *β*-actin expression in (**L**) BPH and (**M**) PCa (scale bar represents 100 *μ*m).

Recent studies have implicated ERK5 signalling in invasive PCa. However, ERK5 expression has not been formally tested in metastatic PCa. Our *in vitro* data would suggest that upregulated ERK5 signalling may contribute to an aggressive cancer phenotype including metastatic disease. Therefore, 32 cases of metastatic prostate tumours were examined for ERK5 immunoreactivity, along with normal prostate, BPH, precursor lesion (PIN) and primary PCa. Both BPH and normal prostate samples expressed ERK5 at similar levels. Consistent with previous data, there was a trend for high levels of cytoplasmic ERK5 expression in tumours with high Gleason sum score (>7) and high serum PSA levels (>10) (data not shown). Importantly, nuclear ERK5 immuno-reactivity in metastatic PCa was significantly stronger than that observed in BPH and primary PCa (*P*=0.013 and *P*<0.0001, respectively, Mann–Whitney test; Figures 4A–D, G and H). Pre-malignant PIN lesions also showed significant upregulation of ERK5 expression, when compared with BPH control (*P*=0.015, Mann–Whitney test; Figure 4E). This would suggest involvement of ERK5 to be an early event in prostate carcinogenesis.

The mechanism for ERK5 activation in PCa remains to be fully examined. MicroRNA mir143 expression has recently been implicated to regulate ERK5 expression (Clape *et al*, 2009). Qualitative analysis of mir143 mRNA and ERK5 protein expression was performed on sequential sections from selected cases of PCa (*n*=3). In keeping with published data, an inverse relationship between mir143 and ERK5 expression was noted, namely reduced mir143 and enhanced ERK5 expression in the malignant epithelium (and vice versa for benign tissue; Figures 4I and J). In addition, mir143 expression was seen in benign prostatic glands, endothelium (Figure 4K) and stromal smooth muscle cells.

Taking together, our data on ERK5 function *in vitro* and *in vivo* as well as expression in clinical PCa support its role in an invasive phenotype.

## Discussion

In this report, we showed the first evidence for upregulated ERK5 expression in metastatic PCa. Upregulated ERK5 expression in PIN lesions would suggest an early involvement of ERK5-mediated signalling in prostate carcinogenesis. Amplification or mutation of the *MEK5* and *ERK5* genes is infrequent; within the data available at catalogue of somatic mutations in cancer (COSMIC, Sanger Institute, UK; www.sanger.ac.uk/genetics/CGP/cosmic), they are relatively uncommon events seen in skin, liver and breast cancer. Our finding of reduced mir143 expression with corresponding enhanced ERK5 expression in sequential tissue sections strongly argue for a role of mir143 at least partly contributing to abnormal ERK5 expression in PCa. Future studies are warranted to validate and define the mechanism for loss of mir143 expression.

Recent data have been accumulating to support a key role of MEK5/ERK5 signalling in carcinogenesis. Although MEK5/ERK5 signalling clearly promotes cell cycle promotion in certain context (Kato *et al*, 1997, 1998), there are situations where ERK5 function does not contribute to proliferation (Squires *et al*, 2002; Wang *et al*, 2005). Abnormal signalling by MEK5/ERK5 has been implicated in a number of tumour types. Amplification of the ERK5 gene locus has been reported in hepatocellular carcinoma where ERK5 function appears to be a key mitogenic factor (Zen *et al*, 2009). In contrast, our data in PCa did not suggest significant mitotic advantage as a result of ERK5 function. Instead, ERK5-mediated signalling appears to critically regulate cellular motility and invasion in PCa, which is in keeping with our observed association between aberrant ERK5 expression in the primary prostate tumours and a less favourable survival outcome (McCracken *et al*, 2008).

Degradation of the ECM is integral to tumourigenesis and is mediated by the activity of ECM proteases, including the MMPs and the serine proteases (Sternlicht and Werb, 2001). There are at least 25 members of the MMP family; their functions include a role in ECM degradation in the context of tumour establishment: growth and migration, avoidance of apoptosis, angiogenesis and interaction with the immune system (Overall and Lopez-Otin, 2002). These effects are achieved in part by the cleavage of growth factors, their receptors, or other growth factor-associated proteins (Egeblad and Werb, 2002). An important means of their inhibition is achieved by the binding of the TIMPs, of which there are four members (Jiang *et al*, 2002).

There is considerable evidence supporting the involvement of MMPs and TIMPs in PCa (Pajouh *et al*, 1991; Trudel *et al*, 2003; Udayakumar *et al*, 2003). Of the known MMPs studied in the prostate, MMP9 overexpression correlates with metastatic disease. Levels of MMP9 mRNA and mature protein have both been shown to be elevated in malignant prostatic tissue, particularly in aggressive and metastatic tumours (Hamdy *et al*, 1994; Wood *et al*, 1997; Dong *et al*, 2001). For the first time, our data implicated expression of MMP12, 16 and TIMP2 to be regulated by ERK5-mediated signalling (Clark *et al*, 2008).

Taking our previous data on MEK5 (Mehta *et al*, 2003) and the current report on ERK5, activation of expression for members of the MMP family, particularly MMP9, by MEK5/ERK5 appear to be an important signalling events. The elements controlling the expression of *MMP9* have been well characterised and include AP-1, NF-*κ*B, Ets and Sp1 sites, mainly within −670-bp region upstream to the transcription start site (Sato and Seiki, 1993). In particular, both the AP-1 and NF-*κ*B-binding sites have been shown to be important regulatory elements of the *MMP9* promoter (Ricca *et al*, 2000; Troussard *et al*, 2000). Serum-induced activation of ERK5 results in MEF2C dependent transcriptional activation of c-jun to increase AP-1 levels (Kato *et al*, 1997).

In this report, we showed that upregulated ERK5 expression promoted MMP expression: (1) enhanced MMP-1, -2 and -9 promoter luciferase reporter activities in PC3 cells (*P*<0.001 for three constructs); (2) MMP and TIMP expression profiling by Q-RT–PCR in HEK293 cells (Supplementary Table 1) (Riddick *et al*, 2005) confirmed increased MMP2 and MMP9 expression (1.8- and 2.1-fold, respectively), although MMP1 mRNA expression was not affected. This apparent difference in MMP1 expression between the promoter reporter assay and the quantitative real time data may be due to the different cell lines used in the two experiments, namely PC3 and 293 cells. Evidence of *in vivo* ECM degradation at the site of invadopodia is interesting. Degradation of gelatin at these sites is consistent with ERK5-mediated upregulated expression of MMP-2 and -9, also referred to as the gelatinase group of MMPs. As MMP12 was significantly upregulated by ERK5 signalling, it is worth noting that MMP12 (or human macrophage elastase) is able to activate MMP2 to exaggerate the cascade of proteolytic processes (Chen, 2004). As our data have suggested effects at the transcription level, future studies to examine the impact of ERK5 signalling on protein expression and functional status of the key MMPs such as MMP9 are required.

Taken together, our *in vitro*, *in vivo* and clinical data support an important role for the MEK5–ERK5 signalling pathway in invasive PCa and may represent a potential target for therapy, including primary and metastatic PCa. In addition, the recent discovery of specific ERK5 inhibitor will provide the molecular probe to further evaluate the molecular basis of ERK5-driven carcinogenesis (Yang *et al*, 2010).
