# Supplementary material for: ERK5 signalling in prostate cancer promotes an invasive phenotype
Source: Br J Cancer. 2011 Jan 25;104(4):664–72. doi: 10.1038/sj.bjc.6606062 (PMC3049582; doi:10.1038/sj.bjc.6606062)
Supplement: Supplementary Information [file 6606062x1.pdf]

MMP (Full Media)	Direction of Change on Induction of MEK5	Fold Change in mRNA Expression on Induction
MMP1	No Change	
**MMP2**	↑	x 1.8
MMP3	N/D	
MMP7	No Change	
**MMP9**	↑	x 2.1
MMP10	No Change	
MMP11	No Change	
**MMP12**	↑	x 2.7
MMP13	No Change	
MMP14	No Change	
MMP15	No Change	
**MMP16**	↓	- x 1.67
MMP17	No Change	
MMP19	-	
MMP20	-	
MMP21	No Change	
MMP22	No Change	
MMP23	No Change	
MMP24	No Change	
MMP25	No Change	
MMP26	-	
MMP27	No Change	
MMP28	No Change	
TIMP1	No Change	
**TIMP2**	↑	x 2.2
TIMP3	No Change	
TIMP4	N/D	

Supplementary table: Summary of expression profiling of MMP genes in response to activation of MEK5-ERK5 pathway The fold change (increase or decrease) in the means between the uninduced and induced groups awere calculated and summarised with a fold increase or decrease of ≥ x1.5 was considered significant. Arrows in the middle column illustrate direction of change in gene expression with overexpression of MEK5. N/D = not detected. The values given are derived from TaqMan expression data after normalisation to 18SrRNA levels, and are probe, and therefore gene-specific.

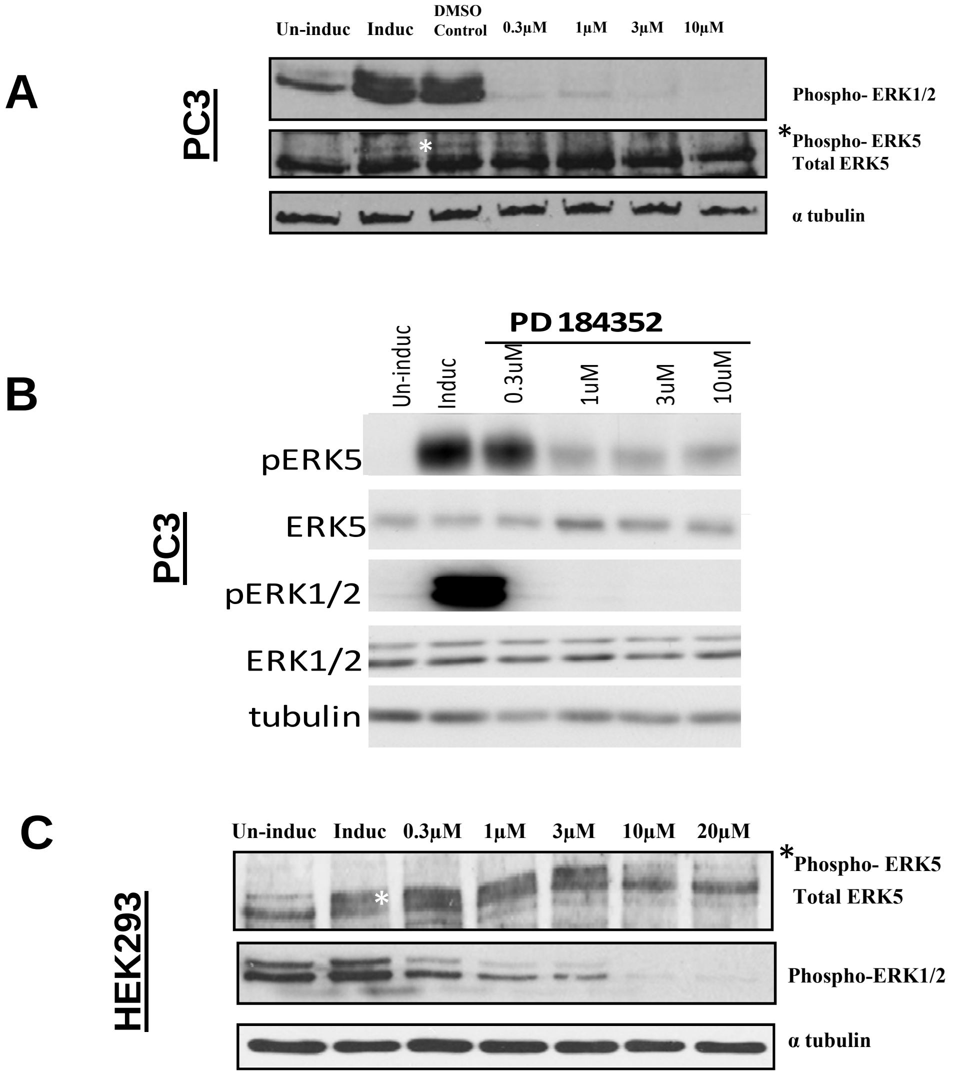

Supplementary figure 1.

Western blots showing differential blockade of ERK1/2 and ERK5 by PD184352 in PC3 and HEK293 stably transfected with MEK5(D) cells.

(A) Validation western blots show differential blockade of ERK1/2 activation alone at lower dose PD184352 (0.3 µM), and blockade of ERK1/2 and ERK5 at higher dose of PD184352 (3 µM) in PC3 cells stimulated with EGF (100 ng/ml)(* signifies band shift associated with phosphor-ERK5).

(B) Similarly, status of ERK5 upon EGF stimulation in the presence of increasing doses of PD184352 was illustrated using specific pERK5 antibody.

(C) Western blot showing differential blockade of ERK1/2 activation alone at lower dose PD184352 (1 µM), and blockade of ERK1/2 and ERK5 at higher dose of PD184352 (10 µM) in MEK5(D) over-expressing HEK293 cell line.

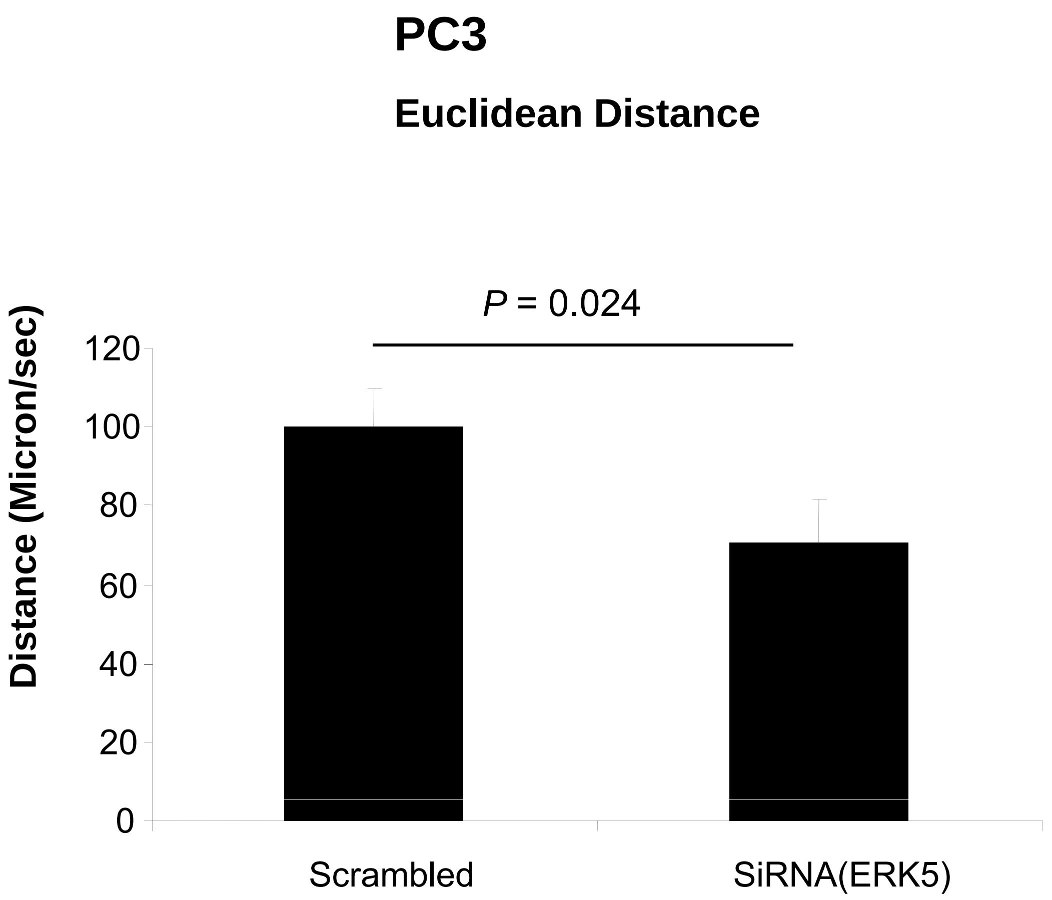

Supplementary figure 2.
siRNA mediated knockdown of ERK5 expression in PC3 resulted in reduced euclidean distance travelled.

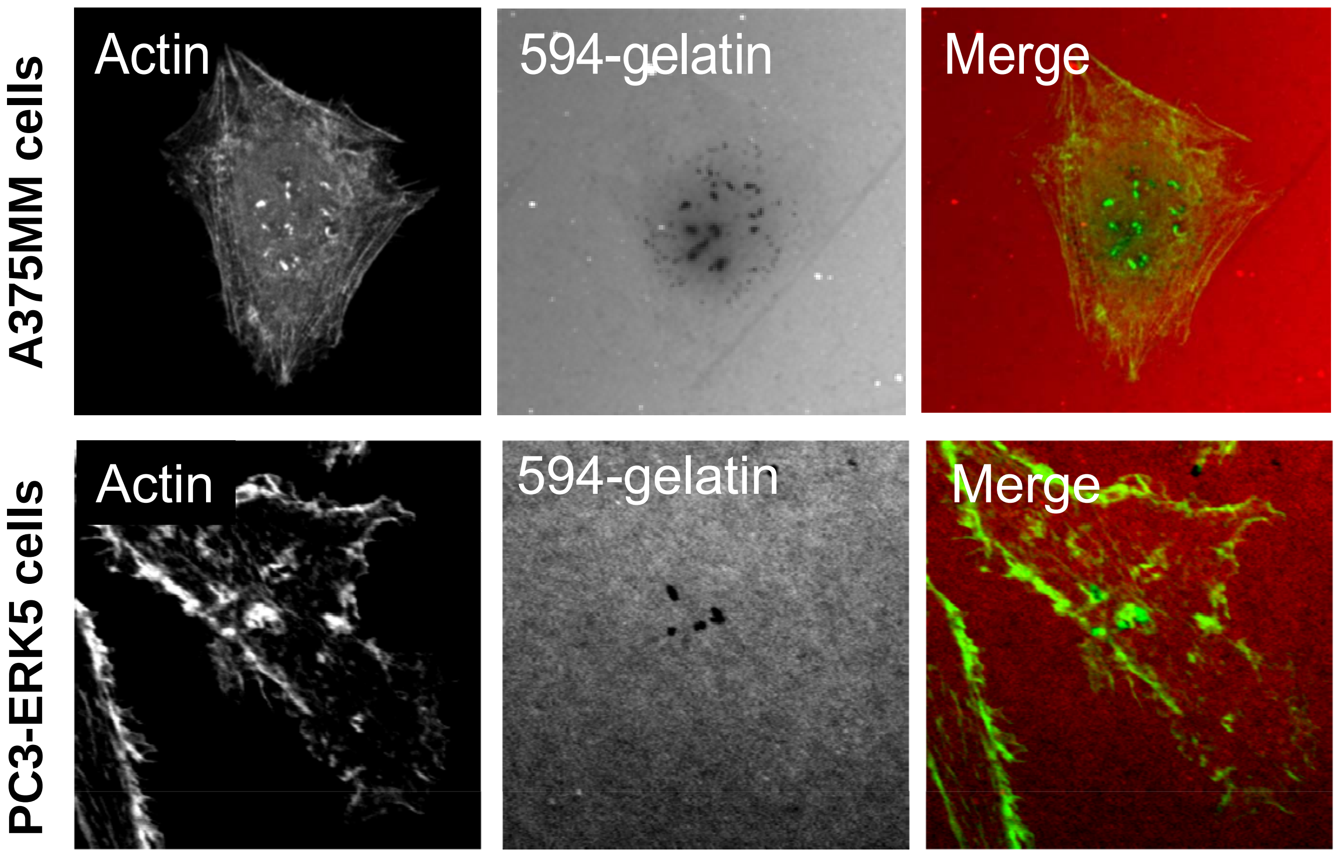

Supplementary figure 3. Invadopodia in A375 melanoma and PC3-ERK5 cells.

A375 or PC3-ERK5 cells were seeded on glass cover slips crossed-linked with 594-gelatin, and leave for 16 hours to allow gelatin degradation. Cells were fixed and stain with 488 phalloidin. Confocal images show actin localization at invadopodia where gelatin degradation occurs.
